# Palaeontological evidence of membrane relationship in step-by-step membrane fusion

**DOI:** 10.3109/09687688.2010.536169

**Published:** 2010-12-29

**Authors:** XIN WANG, WENZHE LIU, KAIHE DU

**Affiliations:** 1 State Key Laboratory of Palaeobiology and Stratigraphy, Nanjing Institute of Geology and Palaeontology, Nanjing; 2 College of Life Sciences, Northwest University, Xi'an; 3 Jiangsu Key Laboratory for Supramolecular Medicinal Materials and Applications, College of Life Sciences, Nanjing Normal University, Nanjing, P. R. China

**Keywords:** Membrane, relationship, fusion, quick, fixing, lightning

## Abstract

Studies on membrane fusion in living cells indicate that initiation of membrane fusion is a transient and hard to capture process. Despite previous research, membrane behaviour at this point is still poorly understood. Recent palaeobotanical research has revealed snapshots of membrane fusion in a 15-million-year-old fossil pinaceous cone. To reveal the membrane behaviour during the fusion, we conducted more observations on the same fossil material. Several discernible steps of membrane fusion have been fixed naturally and observed in the fossil material. This observation provides transmission electron microscope (TEM) images of the transient intermediate stage and clearly shows the relationship between membranes. Observing such a transient phenomenon in fossil material implies that the fixing was most likely accomplished quickly by a natural process. The mechanism behind this phenomenon is clearly worthy of further enquiry.

## Introduction

Membrane fusion is ubiquitous throughout eukary-otic biological systems and has been of great interest in physiological research for decades because it is related to many physiological processes in eukaryotes (Katz 1962, Palade and Bruns 1968, Lucy 1970, Takeo et al. 1973, Heuser et al. 1974, 1979, Chandler and Heuser 1980, Heuser and Reese 1981, Battey et al. 1999, Blatt et al. 1999, Jahn and Südhof 1999, Blatt 2002, Cho et al. 2002, 2004, Yang and Huang 2002, Jahn et al. 2003, Jeremic et al. 2003, Tamm et al. 2003, Taraska et al. 2003, Jahn 2004, Jena 2004, 2005a, 2005b, 2006, 2010, Kelly et al. 2004, Chernomordik and Kozlov 2005, Jahn and Scheller 2006, Siksou et al. 2007, He and Guo 2009, Songer and Munson 2009, Žárský et al. 2009, Lee et al. 2010, Zhang et al. 2010). Membrane fusion is a complicated process involving many enzymes, and many hypotheses have been proposed to explicate it in the past decades (Katz 1962, Palade and Bruns 1968, Lucy 1970, Takeo et al. 1973, Heuser et al. 1974, 1979, Chandler and Heuser 1980, Heuser and Reese 1981, Thiel and Battey 1998, Battey et al. 1999, Blatt et al. 1999, Blatt 2002, Cho et al. 2002, 2004, Jahn et al. 2003, Yang and Huang 2002, Jeremic et al. 2003, Tamm et al. 2003, Taraska et al. 2003, Jahn 2004, Jena 2004, 2005a, 2005b, 2006, 2010, Kelly et al. 2004, Chernomordik and Kozlov 2005, Jahn and Scheller 2006, Takamori et al. 2006, Siksou et al. 2007, Wong et al. 2007, He and Guo 2009, Songer and Munson 2009, Žárský et al. 2009, Lee et al. 2010, Zhang et al. 2010). More and more new technologies have been applied in the related studies and more in-depth understanding of the process has been achieved (Cho et al. 2002, 2004, Yang and Huang 2002, Jahn et al. 2003, Jeremic et al. 2003, Jahn 2004, Jena 2004, 2005a, 2005b, 2006, 2010, Kelly et al. 2004, Chernomordik and Kozlov 2005, Jahn and Scheller 2006, Siksou et al. 2007, Croteau et al. 2009, Songer and Munson 2009, Lee et al. 2010, Zhang et al. 2010). At this time, there still is some gap between hypotheses and observation, however, due to the transience of the initiation of fusion pore that usually takes place in milliseconds, although the whole fusion may last as long as tens of minutes (Cho et al. 2002, 2004, Jena 2006, 2010). Few TEM observations provide direct evidence of details about the intermediate stages and membrane relationship during this process. Wang et al. (2007) report membrane fusion between secretory vesicles and cytoplasmic membrane (CM) in a fossil cone. After more investigation of the same material, we provide more details of the fusion steps and membrane relationship during this transient process, and discuss its implications for biological sample preservation and preparation.

## Materials and methods

The mummified pinaceous cone studied previously by Wang et al. (2007) was further studied here. The cone (PB20715) was collected in 2005 from the Miocene (> 15 million year old) at Clarkia, Idaho, USA (P33, 47°01'N, 116°25'W; Yang et al. 2005) and is now deposited in Nanjing Institute of Geology and Palaeontology, Nanjing, China. Originally it was embedded in a gray siltstone formed in a storm-influenced lake under anoxic conditions (Yang et al. 2005).

**Figure 1:**
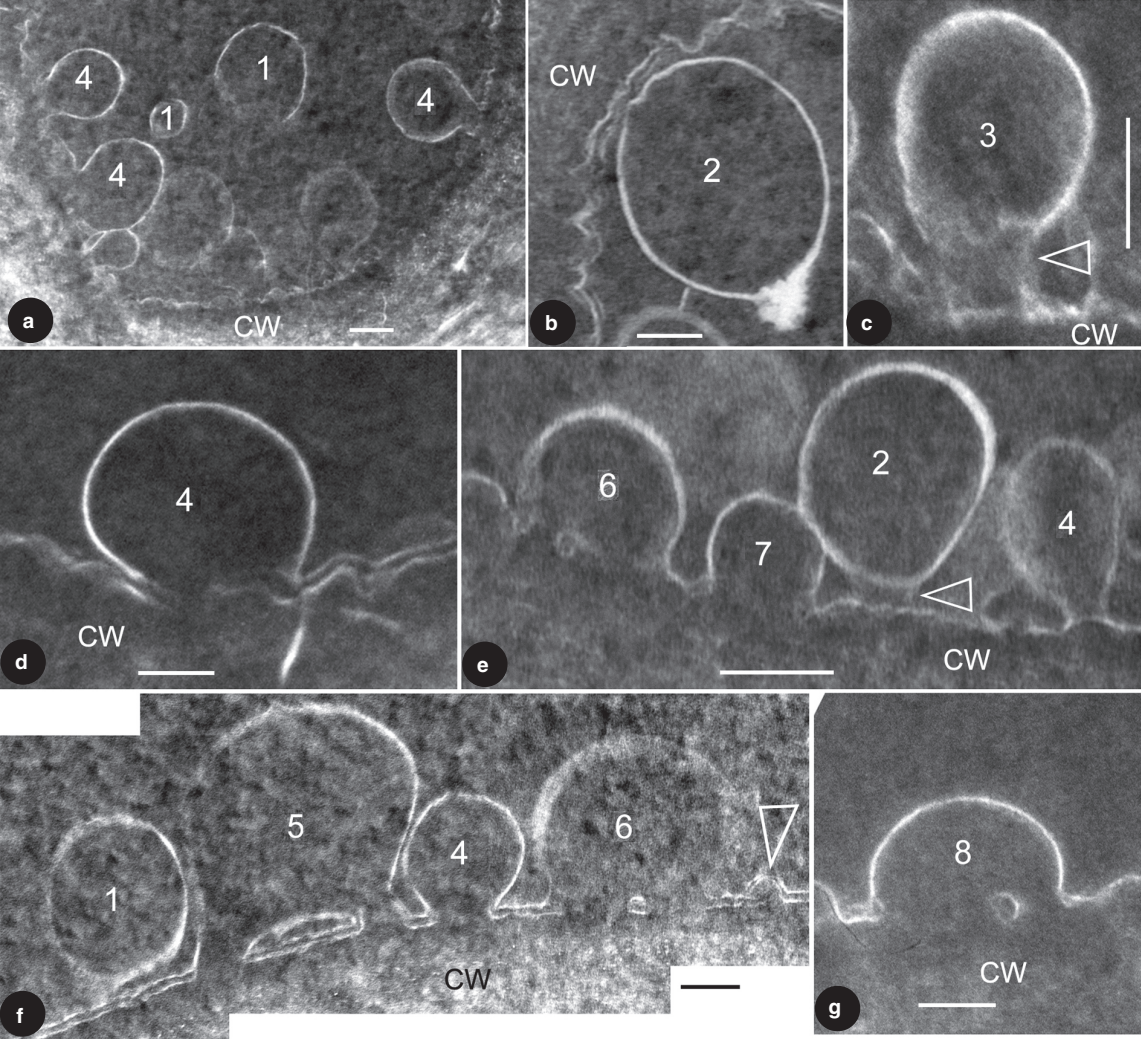
TEM images showing vesicles in various stages of membrane fusion in fossil plant cells. The membrane fusion stages are labeled by numbers. CW, cell wall. Bar= 100 nm. (a) About nine vesicles in various stages of membrane fusion in the same cell. Vesicles in S1 are free from the CM, those in S4 are already fused with the CM. Note the vesicle in the bottom right is blurry while those in the left are sharp. (b) A vesicle near the CM. Note its generally regular spherical form and a slightly distorted region (to the upper left) close to the CM. (c) A vesicle tethered to the CM. Note the cylindrical connection (arrow) between the vesicle and CM. Courtesy of Wang et al. (2007) and MMB. (d) A vesicle fused with the CM. Note the two leaflets of the CM and only one leaflet of the vesicle. The vesicle membrane is connected to the inner leaflet of the CM. Courtesy of Wang et al. (2007) and MMB. (e) Four vesicles in different stages of membrane fusion in the same cell. The vesicle in S2 is only weakly connected to the CM (arrow), the one in S4 is already connected to CM by a narrow neck, the one in S6 is omega-shaped and with a central plug in the fusion opening, and the one in S7 has a wider fusion opening and no visible central plug. (f) Four vesicles in different stages of membrane fusion in the same cell. Note the double layer structure of the CM and its relationship with the membranes of the vesicles. The vesicle in S1 is free from the CM, the one in S5 is already connected to the CM and forms a central plug together with the CM, the one in S6 is connected to the CM and has a relict central plug, and the one in S4 is connected to CM and has no trace of the outer leaflet of the CM. Note all vesicle membranes are connected to the inner leaflet of the CM. Rightmost, there appears to be another vesicle (arrow) with double-leaflet membrane close to its complete fusion with the CM. (g) A vesicle close to complete fusion with CM. Note its shape, relationship with the CM, and a relict plug in the fusion opening.

The fossil was photographed using a Leica MZ-16A stereomicroscope with a digital camera (Wang et al. 2007). Small pieces of the fossilized organic material were taken for microscopic observation, placed sequentially in 20% HCl, 40% HF, and then 20% HCl to remove inorganic minerals. This processing was repeated twice more to ensure demineralization. The samples were embedded in Epon 812 for ultra-thin sections at Nanjing Normal University, Nanjing, China, according to the following procedure. The recipe for the 20 ml resin solution was 10.28 ml Epon 812, 1.24 ml DDSA, 8.48 ml MNA, and 0.34 ml DMP-30. The samples were put in acetate for 3 h, 1 h each in 50%, 67%, and 100% Epon resin solutions in acetate, then in 100% resin solution overnight. Next, samples were immersed in fresh pure Epon resin in a container and cured in a progressively warmer oven set at 30°C for 24 h, 45°C for 24 h, and 60° C for 24 h. Then the cured blocks were trimmed and sectioned using a Leica Ultracut R ultramicrotome set at a 70 nm interval using a diamond knife (Figures 1a-g). The ultrathin sections were stained with lead citrate. Including those in Wang et al. (2007), observations of more than 28 ultrathin sections, each covering more than 100 cells, was performed using an Hitachi-7650 TEM at Nanjing Normal University, Nanjing, China, and a Jeol JEM-1230 electron microscope at Nanjing Institute of Geology and Palaeontology, Nanjing, China. More than hundred vesicles in various stages have been observed more than 475 times. The electron micrographs were saved in TIFF format, and pieced together for publication using Photoshop 7.0.

## Results

The tissues in the cone are well preserved, and dark-stained cells are separated from each other by light-stained cell walls (Wang et al. 2007). Sometimes the double layer structure of CM is evident (Figures 1b, 1d, 1f), but it may disappear due to the discharge of vesicle content (Figures 1a, 1c, 1e, 1g). There may be multiple vesicles of various forms and stages within a single epidermal cell (Figures 1a, 1e, 1f). The vesicles are of various sizes, ranging from 77-383 nm in diameter (Figures 1a-g). The vesicles have a membrane of only one leaflet (Figures 1a-g; Wang et al. 2007). Depending on their positions in the cells, the vesicles have various relationships with the CM and thus can logically be arranged in eight stages (Figures 1a-g). Those in Stage 1 (hereafter abbreviated as S1; the same for other stages) are of spherical form and freely floating in the cytoplasm (Figure 1a). Those in S2 just start interacting with the CM, with their shapes slightly distorted corresponding to that of the CM, compared to those in S1 (Figure 1b). Sometimes the vesicle membrane in S2 appears to have a tendency to interact with the CM (Figure 1e), and this closely resembles that in S3 in term of the blurry connection to the CM. The vesicle in S3 is rarely seen and has its shape slightly stretched, connected to the CM with a cylindrical connection (Figure 1c). This stage may be hard to distinguish from that in S2, such as in Figure 1e. Those in S4 usually have lost their vesicle membrane integration, a loss that already starts in S3. The vesicles in S4 still have their sub-spherical form, connected to the CM openings, sometimes with well established narrow necks, forming a clear omega-shaped configuration (Figures 1a, 1d-f). Vesicles in S4 usually have no clear CM in the fusion opening (Figures 1a, d-f), probably due to the impact of the vesicle content discharge. The vesicle in S5 is rarely seen (Figure 1f). It is omega-shaped and connected to the CM. There are smaller pores on each side of the central plug within the fusion opening on the CM, connecting the interior of the vesicle to the extracellular space (Figure 1f). The central plug is formed by the CM and vesicle membrane, with the vesicle membrane and the inner leaflet of the CM forming a flattened, closed loop (Figure 1f). The vesicles in S6 have wider fusion openings and reduced central plugs (Figures 1e-f). In this stage the CM within the fusion opening, especially the outer leaflet, is hardly visible (Figure 1e). The vesicle in S7 has a still wider fusion opening, and its plug is rarely seen in the fusion opening (Figure 1e). The vesicle in S8 has the widest fusion opening, with a diameter close to that of the vesicle; sometimes the relic of a plug may still be visible (Figure 1g). It is noteworthy that in all case the vesicle membranes interact only with the inner leaflet of the CM, but are never connected to the outer leaflet of the CM.

## Discussion

**Figure 2:**
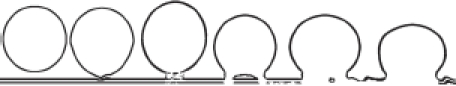
Diagram showing the idealized stages of membrane fusion during an exocytosis.

The Clarkia fossil locality where the studied material was collected is famous worldwide because of its well-preserved fossil plant tissues (Niklas and Brown 1981, Niklas 1982, 1983, Niklas et al. 1985, Golenberg et al. 1990, Soltis et al. 1992, Kim et al. 2004, Collinson et al. 2005). Ultrastructures of nuclei, mitochondria, chloroplasts, starch grains, cell walls, and even DNA sequences have been found in tissues of *Betula, Hydrangea, Platanus, Quercus, Magnolia, Persea,* and *Taxodium* from this locality (Niklas and Brown 1981, Niklas 1982, 1983, Niklas et al. 1985, Golenberg et al. 1990, Soltis et al. 1992, Kim et al. 2004, Collinson et al. 2005). Meanwhile, cytoplasmic relics have been seen in various plant fossils from various regions and ages (Poinar et al. 1996, Schönhut et al. 2004, Wang 2004, 2006, 2007, Koller et al. 2005, Ozerov et al. 2006, Wang and Cui 2007, Wang et al. 2010, in press). The authors (Wang et al. 2007) have reported ultrastructures preserved in the same material studied from the Clarkia site previously. Therefore reporting further ultrastructural details in fossil plant tissues is within expectation and not surprising. This eliminates or reduces the possibility of artifacts in this study. Niklas, Golenberg, Soltis, Kim and their colleagues treat their samples with fixatives, liquid nitrogen, dry ice, or other agents immediately at the fossil site (Niklas and Brown 1981, Niklas 1982, 1983, Niklas et al. 1985, Golenberg et al. 1990, Soltis et al. 1992, Kim et al. 2004, Collinson et al. 2005), assuming this processing helps to keep the fossil stable. However, as all structural biologists know, any processing to one degree or another introduces artifacts. Therefore the question of whether or not the treating introduces artifacts in fossil tissues from the Clarkia site went unanswered until Wang et al. (2007) reported well preserved cells and subcellular structures in untreated material from the same locality. The repeated presence of ultrastructures both in the untreated (Wang et al. 2007; this report) and treated materials (Niklas and Brown 1981, Niklas 1982, 1983, Niklas et al. 1985, Collinson et al. 2005) together strongly suggests that the existence of ultra-structures in these fossil tissues is real and independent of treatment, and that previous treatments did not introduce artifact. The fusing vesicles reported here, and those in Wang et al. (2007), are restricted to the epidermal cells and are completely missing in other cells in the same sections. This contrasting spatial distribution pattern of these vesicles implies that embedding, staining, and other processing in this and previous studies do not produce artifacts because there are none. Any artifact, if it were there, would have left traces over all the sections or tissues and not be restricted to specific cells. Furthermore, a similar phenomenon has been independently reported from a different fossil material much older (30-40 Ma old) and in different preservation (Figure 2d; Poinar et al. 1996). This further reduces the probability that our observation and discussion are based on artifacts. One special feature of fossil materials is that, unlike living materials, fossil materials have been fixed by nature and their fixation is beyond the control of human beings. If a structure is preserved, it must have survived million-year-long diagenesis and has to be stable and lasting; therefore, it usually will not disappear or change in a geologically short time. If no structure is preserved, our processing cannot repeatedly reveal the same specific ultrastructures in the same tissues. Therefore, the authors assume that the ultrastructures reported here that are comparable to those in living materials are not artifacts, but faithfully preserved ultrastructures of the fossil plant.

Membrane fusion plays an important role in many physiological processes in eukaryotes and thus has been a research focus for biological studies for many decades (Katz 1962, Palade and Bruns 1968, Lucy 1970, Takeo et al. 1973, Heuser et al. 1974, 1979, Chandler and Heuser 1980, Heuser and Reese 1981, Blatt et al. 1999, Blatt 2002, Cho et al. 2002, 2004, Yang and Huang 2002, Jahn et al. 2003, Jeremic et al. 2003, Tamm et al. 2003, Taraska et al. 2003, Jahn 2004, Jena 2004, 2005a, 2005b, 2006, 2010, Kelly et al. 2004, Chernomordik and Kozlov 2005, Jahn and Scheller 2006, Siksou et al. 2007, He and Guo 2009, Songer and Munson 2009, Žárský et al. 2009, Lee et al. 2010, Zhang et al. 2010). It is the key event in cell secretion (exocytosis) because this process could not take place without membrane fusion. Generally, exocytosis includes several steps. First, the secretory vesicles move to the periphery of the cell and are guided to the CM. Second, the vesicles approach the CM, their membranes start interacting with the inner leaflet of the CM. Then, through the involve ment of Ca ^+^, enzymes, and proteins, an opening is formed on the vesicle membranes and CM. Finally, part or all the vesicle content is released into the extracellular space (Blatt et al. 1999, Blatt 2002, Jahn et al. 2003, Tamm et al. 2003, Jahn 2004, Jena 2004, 2005a, 2005b, 2006, Jahn and Scheller 2006, He and Guo 2009, Žárský et al. 2009). The mechanism behind this process has been intensively studied recently and many enzymes, membrane proteins, and lipids are found involved in it (Blatt et al. 1999, Blatt 2002, Jahn et al. 2003, Tamm et al. 2003, Jahn 2004, Jena 2004, 2005a, 2005b, 2006, Chernomordik and Kozlov 2005, Jahn and Scheller 2006, Siksou et al. 2007, Croteau et al. 2009, He and Guo 2009, Songer and Munson 2009, Žárský et al. 2009, Jena 2010, Lee et al. 2010, Zhang et al. 2010). The related electron microscopic structural studies on living plant materials were mostly performed between the 1960s to early 1980s (Palade and Bruns 1968, Lucy 1970, Takeo et al. 1973, Heuser et al. 1974, 1979, Chandler and Heuser 1980). Recent progresses are made mainly through applying molecular methods and other new technologies (Cho et al. 2002, 2004, Yang and Huang 2002, Jahn et al. 2003, Jeremic et al. 2003, Jahn 2004, Jena 2004, 2005a, 2005b, 2006, 2010, Kelly et al. 2004, Chernomordik and Kozlov 2005, Jahn and Scheller 2006, Siksou et al. 2007, Croteau et al. 2009, Songer and Munson 2009, Lee et al. 2010, Zhang et al. 2010). Chandler and Heuser (1980) provided details about membrane fusion through TEM observation on samples pre pared by quick freezing. The most detailed documentation of vesicle fusion with CM was done by Palade and Bruns (1968). They used 28 figures to document the various stages of membrane fusion between the vesicles and the CM. Similar omega-shaped vesicles were also reported by Takeo et al. (1973). A recent study on fossil material reveals glances at membrane fusion in plant cells (Wang et al. 2007), similar to that in another fossil material reported by Poinar et al. (1996) in their Figure 2d. These reports are in general agreement with what we report here. What is novel in this report is that the membrane relationships between the vesicles and CM are clearly shown.

Our observations find secretory vesicles in various stages of membrane fusion: isolated in cytoplasm (S1), tethered to CM (S2-S3), and fused with CM (S4-S8) (Figures 1, 2).

Most importantly, the secretory vesicles in the same cell may demonstrate various stages of fusion with the CM (Figure 1a, e-f). The series of vesicles from S1-S6 correspond well with the tethering, docking, hemifusion, and fusion hypothesized by various scholars. One reservation concerns the order between S4 and S5, which may not occur in that order. It is possible that during the membrane fusion S5 actually precedes S4. However, this can neither be confirmed nor rejected based on currently available data. This direct observation of membrane behaviour during membrane fusion using TEM is helpful for determining parameters of this process (Yang and Huang 2002), providing first-hand data for a membrane fusion hypothesis. The so-called intermediate stage in Figures 1 c and 1 e may not be sharp or conspicuous enough. This may be due to one or both of the following reasons. (1) Compared with the stable and well-organized membranes that are distinct and sharp in the images, the tethering structure and the bottom portion of the vesicle membrane are in their reforming and thus unstable, therefore they naturally appear less sharp. Thus the slightly ambiguous image in Figures 1c and 1e may reflect the actual situation in the cell. (2) The sectioning plane may be close to the periphery of the connection and this may also cause the images blurry. A similar situation can be clearly seen in Figure 1 a, in which the vesicles at the bottom right appear blurrier than their peers in the left portion of the same cell. Either of the above may result in blurry image of the so-called connection between the vesicle and CM. However, no matter what the reason is for the blurry appearance, the existence of such structures is a fact and, what is important, this structure has been hypothesized for a long time but has never before been seen with such clarity in living plant tissues.

What is significant is that the vesicle in S5 (Figure 1f) shows the forming of a fusion opening plug, and the relationship between membranes is clearly shown. There are plugs in the central region of the fusion openings in the vesicles in S5, S6, and S8 (Figures 1e-g). These are very similar to the diaphragms seen in the stomata of vesicles during exocytoses in endothelium of rat tissues (Figures 1, 6, 7, 9-16, in Palade and Bruns 1968). The phenomenon has been noted for long time and Palade and Bruns (1968) have described and detailed the fusion opening during membrane fusion. Siksou et al. (2007) also demonstrate hemifusion stages in their Figures 7a, 7c, and 7e. However, they are limited by the resolution of electron tomography and the membrane relationship in the fusion is not shown clearly enough. The membrane fusion seen in this present research is unique in that the vesicle membrane is of single leaflet (Figures 1a-g; Wang et al. 2007) and its relationship between membrane leaflets is more clearly demonstrated (Figures 1b, 1d, 1f). This long-anticipated snapshot lends support to some proposed models for membrane fusion. It appears that the vesicle membrane and the inner leaflet of the CM interact with each other while the outer leaflet plays a less important role during exocytosis. This is plausible considering that, initially, the outer leaflet of the CM is separated from the vesicle by the inner leaflet, which is closer to the vesicle and would naturally play a more important role in the interaction before membrane fusion.

Figure 1f could be interpreted in another different way. The so-called central plug in the fusion opening is not completely isolated from but connected to other vesicle membranes and CM, and this connection is simply not seen in the section. If this is the case, it implies that the central plug is surrounded by multiple smaller pores. This is more compatible with recent research on the structure of porosomes, in which there are several depressions in a pit on the CM (Cho et al. 2002, 2004, Jeremic et al. 2003, Jahn 2004, Jena 2004, 2005a, 2005b, 2006, 2010, Kelly et al. 2004). Ideally, the opening between the vesicle interior and extracellular space can be related to depressions (porosome) in the pit, and central plug to central plug in the pit. However, the currently available evidence is not enough to validate or falsify this interpretation. We hope that future work may shed light on this problem.

Traditional palaeobotanists have had nothing to do with the cytoplasm and ultrastructures within it. However, recent progress in palaeobotany has been repeatedly revealing ultrastructures in fossil plant cells (Poinar et al. 1996, Poinar and Stankiewicz 1999, Wang 2004, 2007, Koller et al. 2005, Ozerov et al. 2006, Wang and Cui 2007, Wang et al. 2010). Various mechanisms have been proposed for the preservation of cytoplasm ultrastructures (Niklas and Brown 1981, Niklas 1982, 1983, Niklas et al. 1985, Poinar et al. 1996, Schönhut et al. 2004, Wang 2004, 2007, Koller et al. 2005, Ozerov et al. 2006). One of them, proposed by Wang (2004), is lightning fixation although it remains hypothetical due to a lack of supporting evidence. Our observations, together with similar independent observation by Poinar et al. (1996) in their Figure 2d (although otherwise inter preted originally), implies that membrane fusion, a quick process requiring energy from a living cell to overcome electrostatic repulsion between two opposing membranes (Jahn 2004, Chernomordik and Kozlov 2005, Wong et al. 2007), can be fixed by a natural process. Although Heuser et al. (1974) have shown partial fusion events at frog neuromuscular junctions, the membrane relationship in the intermediate stage remain elusive. Exocytosis may last as long as 10 or more minutes (Cho et al. 2002, 2004; Jena 2006, 2010), but studies show that the initiation of a fusion opening usually takes place in a few milliseconds (Monck and Fernandez 1996; Chernomordik and Kozlov 2005). This explains the lack of sharp images showing membrane relationship at the instant of fusion opening forming in spite of much effort invested and many advanced technologies applied (Katz 1962, Palade and Bruns 1968, Lucy 1970, Takeo et al. 1973, Heuser et al. 1974, Chandler and Heuser 1980, Heuser and Reese 1981, Blatt et al. 1999, Blatt 2002, Cho et al. 2002, 2004, Jeremic et al. 2003, Jahn et al. 2003, Tamm et al. 2003, Taraska et al. 2003, Jahn 2004, Jena 2004, 2005a, 2005b, 2006, 2010, Kelly et al. 2004, Chernomordik and Kozlov 2005, Jahn and Scheller 2006, Siksou et al. 2007, He and Guo 2009, Songer and Munson 2009, Žárský et al. 2009, Lee et al. 2010, Zhang et al. 2010). Since the quick freezing technique, which completes the fixation within 2 milliseconds (Heuser et al. 1979, Chandler and Heuser 1980), has been missing this snapshot, it is reasonable to assume that the natural process capturing this snapshot in the fossil materials must be very brief. The only currently available candidate process with sufficient power is lightning, which has a strong microwave radiation that can kill plants, can reach a temperature of up to 36,000°K within 10 microsec (Uman 1969). This proposal is in line with the study on microwave fixation technology, which alone may fix biological samples in distilled water (Login and Dvorak 1988). The bottom line is that, even if fixation takes tens of minutes, it is still a rapid process in geological terms. Before more information is available proving that other candidate processes have such a potential, we assume that lightning most likely is responsible for this quick fixing. However, whether or not this is true is hinged on a study of the effects of lightning on living plants, which unfortunately is still lacking.

## Conclusions

The membrane behaviour during the membrane fusion has been fixed by nature in a fossil material. Whatever the process responsible for the fixation, it is clear that nature has done a superior job of fixation. Studying the natural mechanism behind this phenomenon will help to improve our technique of preparing good and lasting biological samples.
